# A Novel Edge Cache-Based Private Set Intersection Protocol via Lightweight Oblivious PRF

**DOI:** 10.3390/e25091347

**Published:** 2023-09-16

**Authors:** Jing Zhang, Li Yang, Yongli Tang, Minglu Jin, Shujing Wang

**Affiliations:** College of Software, Henan Polytechnic University, Jiaozuo 454000, China

**Keywords:** private set intersection, edge computing, multi-party cooperative cache, concrete efficiency

## Abstract

With the rapid development of edge computing and the Internet of Things, the problem of information resource sharing can be effectively solved through multi-party collaboration, but the risk of data leakage is also increasing. To address the above issues, we propose an efficient multi-party private set intersection (MPSI) protocol via a multi-point oblivious pseudorandom function (OPRF). Then, we apply it to work on a specific commercial application: edge caching. The proposed MPSI uses oblivious transfer (OT) together with a probe-and-XOR of strings (PaXoS) as the main building blocks. It not only provides one-sided malicious security, but also achieves a better balance between communication and computational overhead. From the communication pattern perspective, the client only needs to perform OT with the leader and send a data structure PaXoS to the designated party, making the protocol extremely efficient. Moreover, in the setting of edge caching, many parties hold a set of items containing an identity and its associated value. All parties can identify a set of the most frequently accessed common items without revealing the underlying data.

## 1. Introduction

Co-creation and sharing gained significance in the transition from the era of information technology to the era of digital technology. While information sharing brings convenience, the risk of privacy breaches also rises. The private set intersection (PSI) protocol is a widely used approach to distributed set computation. It is devoted to the joint intersection calculation of data from two or more parties. The PSI protocol guarantees that all parties can collaboratively calculate the intersection of the sets without disclosing anything beyond that intersection. PSI plays an important role in improving pattern matching [1], private contact discovery [2], advertisement conversion rate [3], and edge caching [4]. Edge caching is a key technology for communication networks. In order to utilize cache resources more efficiently, individual operators tend to keep their public items in a shared cache that can be accessed by all parties. However, since the cache is shared among multiple parties, these parties aim to identify the set of most frequently visited common data items and add them to the network edge cache. Their objective is to achieve this without revealing the actual underlying data. This is known as the multi-party shared cache problem, where determining the common term is a typical private set intersection problem.

Most of the current efficient PSI protocols are built on OT [5,6,7]. The OT-based PSI protocols offer greater advantages in terms of communication and computation when compared with PSI based on public key encryption [8,9] and PSI based on a garbled circuit [10,11,12]. Efficient OT extension techniques allow parties to generate many OT protocol instances at a low computational cost through a few public key operations. Chase et al. [5] implemented a two-party PSI protocol with one-sided malicious security. This protocol uses OT and a multi-point OPRF to achieve a good balance between computational and communication overhead. The protocol can only interact between two parties, and multiple runs are required to accomplish the intersection computation with multiple parties involved. Kavousi et al. [13] proposed a MPSI based on OT and multi-point OPRF. This protocol can only be implemented in the semi-honest model. Inbar et al. [14] presented an enhanced semi-honest MPSI protocol based on OT and a garbled Bloom filter (GBF). However, the protocols [13,14] require the transmission of the GBF for communication, creating a certain degree of communication burden.

In response to the above issues, we constructed an MPSI protocol for malicious actors that combines a PaXoS and multi-point OPRF based on OT. The protocol relies only on symmetric keys, hashing, coding techniques, and bitwise operations, thus providing good computational performance. This protocol can solve the problem of privacy-preserving edge cooperative cache sharing by making a simple transformation of this protocol. We show the following contributions:

- Multi-party PSI protocol: We propose a specifically efficient MPSI protocol utilizing OT and a PaXoS. The PaXoS can be seen as a corresponding Encode/Decode algorithm achieving a constant rate. Therefore, our protocol has good computational performance. The protocol has low communication overhead since the clients only need to send a data structure. Theoretical analysis shows that the protocol leads to a better balance between communication and computational cost.
- Security against malicious clients: We present that our protocol uses the data structure PaXoS to hide the key during encoding to resist malicious adversaries, which can achieve one-sided malicious security against the clients with almost no additional overhead. At the same time, we prove that the protocol can also resist any possible collusion attack from malicious clients.
- Multi-party cooperative cache: Our MPSI protocol can be applied to edge caching scenarios by using cuckoo hashing and simple hashing. The protocol supports having data associated with each input and the extension of payloads to multi-party. In a multi-party cooperative cache (MPCCache) setting, the MPCCache protocol allows parties to compute a sum depending on the data associated with the intersection items. Compared with [4], our MPCCache protocol eliminates the computing burden associated with polynomial interpolation and improves computational efficiency.

## 2. Related Work

**PSI.** The development of efficient constructions for PSI functionality has received considerable research attention in the last decade or more. Some of the recent relevant works on PSI are illustrated in Table 1. Ghosh et al. [15] presented a MPSI protocol using oblivious linear function evaluation (OLE) with optimal asymptotic communication complexity. However, the balance between communication and computation cost is not good. Kolesniko [16] proposed a two-party PSI protocol against semi-honest adversaries. The protocol is mainly based on OT techniques for security string equivalence testing and is computationally efficient. Pinkas [17] proposed a two-party semi-honest PSI protocol based on OT and a GBF. The parallelized processing of the protocol allows for some improvement in protocol efficiency. Nevo [18] proposed a malicious PSI protocol utilizing oblivious programmable PRF (OPPRF) and oblivious key-value store (OKVS) technology, which solves the problem of multi-party PSI against malicious adversaries. However, this protocol does not lead to a better trade-off between communication and computational overhead. Pinkas [19] also proposed a PSI protocol for two parties in the malicious model which uses a PaXoS to implement, for the first time, a malicious secure PSI using cuckoo hashing. Ben-Efraim et al. [20] implemented malicious MPSI based on a GBF for multiple parties. However, GBFs suffer from a certain false positive rate and their high communication overhead. Bui et al. [21] constructed an optimized semi-honest PSI based on a pseudorandom correlation generator (PCG). Additionally, they can use the PCG to construct protocols with fully malicious security in the standard model.

**Function-based PSI.** Many studies have focused on developing efficient techniques for PSI construction. In addition, these studies have explored the output results of computing a function over intersections, allowing for potential extensions to various business scenarios. Table 2 shows recent related works on function-based PSI. Ion et al. [3] proposed a PI-Sum Protocol utilizing Diffie–Hellman (DDH) and homomorphic encryption (HE). Thinking about the advertising (Ad) conversion problem: Ad providers want to analyze Ad effectiveness by age, which obviously cannot be solved using the PI-Sum. Chida [22] proposed a new function based on OPRF and DDH assumptions to calculate the weighted sum of two-party privacy sets (PIW-sum), which has more practical application value. Pinkas et al. [11] proposed an idea of calculating payloads based on the circuit, OPPRF, and cuckoo hash constructs, which allows each input item from one party to have payload data attached to it, and finally to calculate some specific functions of the payloads in the intersection set. Based on a new shuffled distributed oblivious PRF (DOPRF), Miao et al. [23] constructed a two-party PSI cardinality (PSI-CA) protocol for malicious settings which achieves a good computation and communication cost. In the above protocols, only one party can own the payload data, which can be applied in limited practical scenarios. Nguyen et al. [4] extended payload data to the multi-party setting and proposed an MPCCache sharing framework based on polynomial interpolation and OPPRF, which enables multiple parties to calculate a sum of data payloads on each of common data items and can identify the most frequently accessed data items.

## 3. Preliminaries

### 3.1. Notions

The computational and statistical security parameters are denoted by λ and σ. [n] stands in for the set {1,…,n}. $\overset{R}{\leftarrow }$ indicates uniformly random selection. The notation || denotes concatenation between strings. ${\left\{0,1\right\}}^{*}$ denotes the set of strings consisting of 0 and 1, where * means that the strings in the set can be of any length. We use $\overset{C}{\approx }$ to indicate that the real world is indistinguishable from the ideal world. We denote with v[i] the *i*-th element of a vector v of length l. The *i*-th column vector i∈[n] of the matrix $M_{n\times m}$ is denoted by the symbol $M_{i}$. The Hamming weight of the binary string x is represented by $||x|{|}_{H}$.

### 3.2. One-Sided Malicious Security

One-sided malicious security [5] is a security property found in cryptographic protocols wherein one party is allowed to engage in arbitrary malicious behavior in an attempt to compromise security while the other parties follow specified behavioral guidelines. In this context, only the targeted party is vulnerable to malicious action, whereas the other parties maintain their assigned roles and responsibilities. Our MPSI protocol achieves unilateral malicious security against the clients, as they are considered as a whole. We further prove that the proposed MPSI is secure against malicious clients.

### 3.3. Security Model

**Figure 1:**
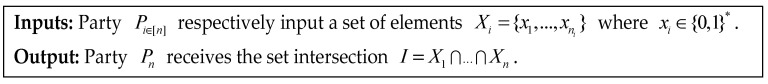
Ideal functionality of MPSI $F_{\mathrm{MPSI}}$.

MPSI is a unique instance of secure multi-party computation (MPC). We adhere to the MPC standard security definition. The ideal functionality of MPSI is defined in Figure 1.

The security models [24] of secure multi-party computation are divided into semi-honest and malicious models. For the semi-honest model, an adversary can completely obey the protocol execution process, yet might record all the data in the protocol execution process and try to learn more from the data generated during the protocol execution process. The adversary under the malicious model can not only infer the sensitive information through the data of the protocol process but also refuse to participate in the protocol, alter the private input set information, or prematurely stop the protocol from running. Our protocol can achieve one-sided malicious security.

**Definition** **1.** *(Malicious security against the clients) If there is a PPT adversary* A *who might unilaterally depart from the protocol in the real world, there exists a PPT adversary* S *who could modify the input to the ideal functionality and terminate the output in an ideal world. Then, the protocol Π can protect from malicious clients, such that for each input* $X_{1},\ldots ,X_{n}$:

(1) $${\mathrm{Real}}_{A}^{\prod }\left(X_{1},\ldots ,X_{n}\right)\overset{c}{\approx }{\mathrm{Ideal}}_{S}^{F}\left(X_{1},\ldots ,X_{n}\right).$$

### 3.4. Oblivious Transfer

**Figure 2:**
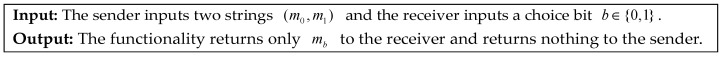
Ideal functionality of OT $F_{\mathrm{OT}}$.

Rabin et al. [25] proposed a crucial cryptographic primitive OT. In a 1-out-of-2 OT configuration, the receiver can have a choice bit b∈{0,1}, while the sender can have input strings $\left(m_{0},m_{1}\right)$. The OT acts to prevent the receiver from knowing nothing regarding $m_{1-b}$ and prevent the sender from learning anything about b. OT necessitates costly public-key operations. Ishai et al. [26] described an OT extension technique that permits many OT executions at the cost of doing few public-key procedures. We can use the instantiation OT in [15]. The ideal functionality of OT is defined in Figure 2.

### 3.5. PaXoS

The following is a way to encode key-value mapping into a brief data structure using a PaXoS [19]. The associated Encode/Decode methods are frequently more convenient to describe when describing a PaXoS than the u mapping.

$\mathrm{Encode}((x_{1},y_{1}),\ldots ,(x_{t},y_{t}))$: Given t items $\left(x_{i},y_{i}\right)$, where $x_{i}\in {\left\{0,1\right\}}^{*}$ and $y_{i}\in {\left\{0,1\right\}}^{w}$, indicate via M the t×m matrix where the *i*-th row is $u(x_{i})$. Note that u(x) is the result of using the mapping u to x. It is possible to find a data structure (matrix) $D={\left(d_{1},\ldots ,d_{m}\right)}^{T}\in {\left({\left\{0,1\right\}}^{w}\right)}^{m}$ satisfying $M\times D={\left(y_{1},\ldots ,y_{t}\right)}^{T}$. In particular, the subsequent linear system of equations is fulfilled when the $u(x_{i})$’s are linearly independent:

(2) $$\left[\begin{array}{c} -u(x_{1})-\\ -u(x_{2})-\\ \vdots \\ -u(x_{t})- \end{array}\right]\times \left[\begin{array}{c} d_{1}\\ d_{2}\\ \vdots \\ d_{m} \end{array}\right]=\left[\begin{array}{c} y_{1}\\ y_{2}\\ \vdots \\ y_{t} \end{array}\right].$$

Decode(D,x): Given $D\in {\left({\left\{0,1\right\}}^{w}\right)}^{m}$ and $x\in {\left\{0,1\right\}}^{*}$, we can extract the corresponding “value” via $y=\langle u(x),D\rangle \overset{\mathrm{def}}{=}\underset{j:v{\left(x\right)}_{j}=1}{⊕}d_{j}$.

### 3.6. Multi-Point OPRF

Chase [5] presented a PSI protocol for two parties based on multi-point OPRF. The sender chooses a pseudorandom seed $s\overset{R}{\leftarrow }{\left\{0,1\right\}}^{w}$, and the receiver computes a pseudorandom function $v=F_{k}(x_{i})$ based on its set elements to construct two matrices: $A_{m\times w}$ and $B_{m\times w}$. For each $x_{i}\in X_{1}$, the corresponding bits in matrices are the same, while others are different. The sender obtains a matrix $C_{m\times w}$ depending on seed s and runs w OTs with the receiver. Each column of the matrix is either $A_{j}$ or $B_{j}$ for all j∈[w]. Then, the sender computes $v=F_{k}(x_{i})$ according to each element $x_{i}\in X_{2}$ to obtain all the resulting OPRFs $\varphi =H\left(C_{1}[v[1]||\ldots ||C_{w}[v[w]]\right)$ and sends them to the receiver. Eventually, the receiver can find the intersection of the two sets based on its computed OPRF value.

### 3.7. Hamming Correlation Robustness

Under the assumption of correlation robustness for the underlying hash function, our MPSI structure is demonstrated to be secure.

**Definition** **2.** *(Hamming Correlation Robustness [5]) If the distribution produced by the sampling of* $s\leftarrow {\left\{0,1\right\}}^{n}$ *at random is pseudorandom for* $a_{1},\ldots ,a_{m}$*,* $b_{1},\ldots ,b_{m}\in {\left\{0,1\right\}}^{n}$*, and has* $||b_{i}|{|}_{H}\geq d$ *for each* i∈[m]*,* H *is d-Hamming correlation robust. Namely:*

(3) $$\left(H\left(a_{1}⊕[b_{1}\cdot s]\right),\ldots ,H\left(a_{m}⊕[b_{m}\cdot s]\right)\right)\overset{c}{\approx }\left(F\left(a_{1}⊕[b_{1}\cdot s]\right),\ldots ,F\left(a_{m}⊕[b_{m}\cdot s]\right)\right),$$

*where* ⊕ *denotes bitwise-AND and bitwise-XOR, respectively, and* F *is a random function.*

### 3.8. Cuckoo Hashing and Simple Hashing

Hash technology is one of the essential tools for optimizing communication and computational complexity in PSI protocols. There are two commonly used construction methods for hash technology: simple hashing and cuckoo hashing [10]. Simple hashing can map elements to k positions in a hash table using k hash functions, with each bucket being capable of storing multiple elements. Cuckoo hashing can map elements to a specific location in a hash table using a hash function, and its basic idea is to use multiple hash functions to handle collisions. When collisions occur, cuckoo hashing evicts the element occupying the original position, which can be rehomed to alternative positions. If alternative positions are already occupied, the process repeats until all elements can find their homes. Typically, cuckoo hashing and simple hashing are combined to achieve optimal results in PSI protocols.

## 4. Our MPSI Protocol

### 4.1. Overview

**Figure 3:**
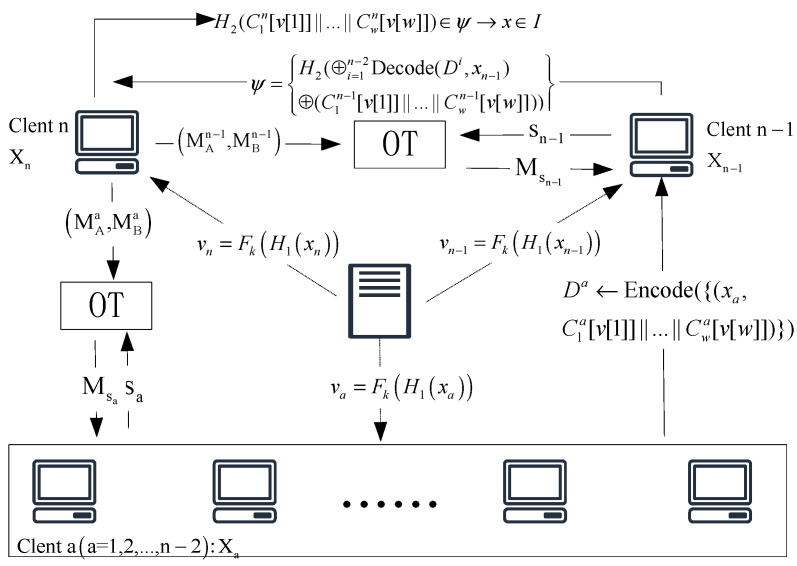
System model.

In this section, we show the MPSI protocol. A couple of parties $P_{1},\ldots ,P_{n}$ with respective private input sets $X_{1},\ldots ,X_{n}$ desire to collectively compute $X_{1}\cap \ldots \cap X_{n}$ without disclosing any more information. Note that we regard t as the set sizes for parties, $P_{n}$ as the leader, and $P_{i\in [n-1]}$ as the client. The system model of the MPSI protocol is shown in Figure 3.

$P_{n}$ constructs a random matrix $A_{m\times w}$ and chooses strings for $P_{i\in [n-1]}$ to generate the $A_{j}^{i}\overset{R}{\leftarrow }{\left\{0,1\right\}}^{m}$ and sets $A_{j}^{n}=A_{j}^{1}⊕\ldots ⊕A_{j}^{n-1}$, where j∈[w]. For each i∈[n−1], from its input elements, $P_{n}$ constructs unique matrices $B_{m\times w}$. $P_{n}$ first initializes a matrix $E_{m\times w}$ to all 1’s. For $x\in X_{n}$ computing $v=F_{k}(H_{1}(x))$, $B_{m\times w}$ is designed such that $E_{j}[v[j]]=0$ for all j∈[w], and hence $A_{j}^{i}[v[j]]=B_{j}^{i}[v[j]]=C_{j}^{i}[v[j]]$ for all i∈[n−1] and j∈[w]. Then, $P_{i\in [n-2]}$ locally encode a data structure PaXoS of their input sets $D^{i}\leftarrow \mathrm{Encode}\left(\left\{\left(x,C_{1}^{i}[v[1]]||\ldots ||C_{w}^{i}[v[w]]\right)\right\}\right)$ using the entries of the received matrix and send $D^{i}$ to $P_{n-1}$. $P_{n-1}$ decodes all the $D^{i}$. Then, they compute and sends the OPRF values $\varphi =H_{2}\left({⊕}_{i=1}^{n-2}\mathrm{Decode}(D^{i},x)⊕\left(C_{1}^{n-1}[v[1]]||\ldots ||C_{w}^{n-1}[v[w]]\right)\right)$ to $P_{n}$. After receiving the OPRF values, $P_{n}$ computes $\varphi =H_{2}\left(C_{1}^{n}[v[1]]||\ldots ||C_{w}^{n}[v[w]]\right)$ according to its input set, which allows $P_{n}$ to find the intersection. This implies that, if x∈I, the hash function’s input from $P_{n-1}$ and $P_{n}$ will be equal. While the output of the PRF would be pseudorandom to $P_{n}$ if x∉I, the hash function’s input from $P_{n-1}$ will be dramatically different from any $P_{n}$’s input.

### 4.2. Our Protocol

**Figure 4:**
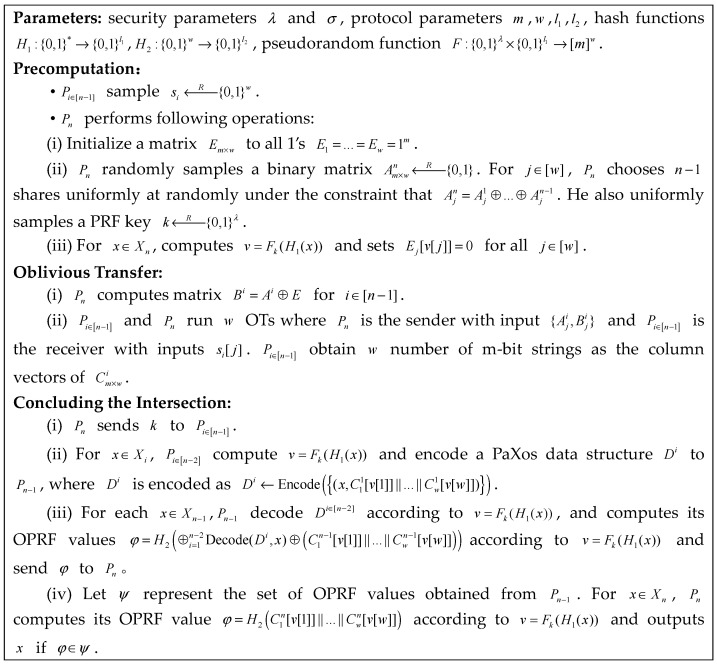
Our MPSI protocol.

We show our MPSI protocol in Figure 4. The selection of m, w, $l_{1}$, and $l_{2}$ in our MPSI protocol follows [5] and they show how to choose the parameters concretely.

### 4.3. Protocol Correctness

$P_{n}$ constructs the special matrices $A^{i}$ and $B^{i}$ for $P_{i\in [n-1]}$ such that $v=F_{k}(H_{1}(x))$ computed for each $x\in X_{n}$ satisfies $A_{j}^{i}[v[j]]=B_{j}^{i}[v[j]]$ for all j∈w. Let x be the intersection element. Since each column of matrix $A_{j}^{n}$ is composed of uniform random shares as $A_{j}^{n}=A_{j}^{1}⊕\ldots ⊕A_{j}^{n-1}$ for j∈w, after the client $P_{i\in [n-1]}$ runs OTs with $P_{n}$, the matrix $C_{j}^{i}$ is obtained, satisfying $A_{j}^{i}[v[j]]=C_{j}^{i}[v[j]]$. It holds that $A_{j}^{n}[v[j]]={⊕}_{i=1}^{n-1}C_{j}^{i}[v[j]]$ for each x∈I.

Based on the nature of the constructed data structure, we have $\mathrm{Decode}(D^{i},x)=C_{1}^{i}[v[1]]||\ldots ||C_{w}^{i}[v[w]]$. So, for x∈I, let $v=F_{k}(H_{1}(x))$, and we can always satisfy $H_{2}\left({⊕}_{i=1}^{n-1}\left(C_{1}^{i}[v[1]]||\ldots ||C_{w}^{i}[v[w]]\right)\right)=H_{2}\left(A_{1}^{i}[v[1]]||\ldots ||A_{w}^{i}[v[w]]\right)$.

### 4.4. Protocol Security

**Theorem** **1.** *If* F *is a PRF,* $H_{1}$ *and* $H_{2}$ *are random oracles, and the underlying OT is protected against malicious receivers, then our MPSI protocol has one-sided malicious security which can be secure against malicious clients when* m, w*,* $l_{1}$*, and* $l_{2}$ *are chosen appropriately.*

**Proof** **of** **Theorem** **1.** We consider any client $P=\{P_{1},\ldots ,P_{n-1}\}$ corrupted by an adversary A. Let l clients $P_{1},\ldots ,P_{l}$ be corrupted, making the number of uncorrupted clients (n−l−1). Given ${\left\{X_{i}\right\}}_{i\in [l]}$, the simulator S interacts with ${\left\{P_{i}\right\}}_{i\in [l]}$ as follows. S samples random matrices ${\left\{C_{i}\right\}}_{i\in [l]}\in {\left\{0,1\right\}}^{m\times w}$ and performs malicious OT simulator on ${\left\{P_{i}\right\}}_{i\in [l]}$ with outputs $C_{1}^{i},\ldots ,C_{w}^{i}$. S honestly chooses PRF key k and sends k to ${\left\{P_{i}\right\}}_{i\in [l]}$. The simulator S constructs random data structures representing honest parties according to the randomness of the matrices. $T_{1}$ and $T_{2}$ are initialized to an empty table. In $P_{i\in [n-1]}$’s query x to $H_{1}$, S records $\left(x,H_{1}(x)\right)$ in table $T_{1}^{i}$. In $P_{n-1}$’s query y to $H_{2}$, S records $\left(y,H_{2}(y)\right)$ in table $T_{2}$. When $P_{n}$ receives OPRF value Ψ, S finds all φ∈Ψ such that $\varphi =H_{2}(y)$ for some y in $T_{2}$, and $y={⊕}_{i\in [l]}\left(C_{1}^{i}[v[1]]||\ldots ||C_{w}^{i}[v[w]]\right)⊕\left({⊕}_{j\in [n-t-1]}\left(C_{1}^{j}[v[1]]||\ldots ||C_{w}^{j}[v[w]]\right)\right)$ where $v=F_{k}(H_{1}(x))$ for $x\in T_{1}^{1}\cap \ldots \cap T_{1}^{n-1}$. Finally, S can send these x to ideal functionality.

Let $Q_{1}^{i},Q_{2}$ be a set of queries $P_{i\in [n-1]}$ and $P_{n-1}$ make to $H_{1}$ and $H_{2}$, respectively, and let $Q=\cap_{i=1}^{n-1}Q_{1}^{i}$, $Q_{1}^{i}=|Q_{1}^{i}|$, and $Q_{2}=|Q_{2}|$. We will misuse notation: for matrix $C_{m\times w}$ and vector $u\in {\left[m\right]}^{w}$, C[v] means $C_{1}[v[1]]||\ldots ||C_{w}[v[w]]$. For the set V of vectors in ${\left[m\right]}^{w}$, the set $\left\{C[V]|v\in V\right\}$ is denoted by C[V].

We prove ${\mathrm{Real}}_{A}^{\prod }\left(X_{1},\ldots ,X_{n}\right)\overset{c}{\approx }{\mathrm{Ideal}}_{S}^{F}\left(X_{1},\ldots ,X_{n}\right)$.

Hyb0 The outputs of parties in the real world.
Hyb1 Similar to Hyb0, but S performs OT simulator on ${\left\{P_{i}\right\}}_{i\in [l]}$ to obtain $s_{i}$. If $s_{i}[j]=0$, it randomly chooses string $A_{j}^{i}$ of length m and constructs matrix $B_{j}^{i}=A_{j}^{i}⊕D_{j}$, and it randomly chooses string $B_{j}^{i}$ of length m and constructs matrix $A_{j}^{i}=B_{j}^{i}⊕D_{j}$; otherwise, it gives $C_{1}^{i},\ldots ,C_{w}^{i}$ to OT simulator as output. Hyb1 is computationally indistinguishable from Hyb0 due to OT security against malicious receiver.
Hyb2 Similar to Hyb1 except that the protocol terminates if there exists $x^{a},x^{b}\in X_{1}\cup X_{2}\cup \ldots \cup X_{n}$, $x^{a}\neq x^{b}$ such that $H_{1}(x^{a})=H_{1}(x^{b})$. Since $H_{1}$ is a random oracle, the protocol is aborted with negligible probability.
Hyb3 Same as Hyb2, but, for each OPRF value φ received by $P_{n}$, if $\varphi \notin H_{2}(Q_{2})$, then $P_{n}$ ignores φ. Since $H_{2}$ is a random oracle, the probability of changing $P_{n}$’s output is negligible. φ equals the output of $H_{2}$ on one of $P_{n}$’s elements with negligible probability.
Hyb4 Same as Hyb3 except that the protocol terminates if there exists $y\in Q_{2}$, $y^{\prime }\in A\left[F_{k}(H_{1}(X_{n}))\right]$ with $y\neq y^{\prime }$ and $H_{2}(y)=H_{2}(y^{\prime })$. Since $H_{2}$ is a random oracle, the protocol is aborted with negligible probability.
Hyb5 Same as Hyb4, but, for each OPRF value φ received by $P_{n}$, $P_{n}$ ignores φ when calculating the set intersection if $\varphi =H_{2}(y)$ for some $y\in Q_{2}$, where $y\notin \left({⊕}_{i\in [t]}\left(C^{i}\left[F_{k}(H_{1}(Q))\right]\right)\right)⊕\left({⊕}_{j\in [n-t-1]}\left(C^{j}\left[F_{k}(H_{1}(Q))\right]\right)\right)$.
  This hybrid changes output only if there exist $x\in X_{n}$ satisfying $\varphi =H_{2}\left(A\left[F_{k}(H_{1}(x))\right]\right)$, which implies $y=A\left[F_{k}(H_{1}(x))\right]$ via the terminate condition added in Hyb4.
  Note that if $x\in X_{n}$ and x∈Q, because of the construction of E, we then have $y=A\left[F_{k}(H_{1}(x))\right]={⊕}_{i\in [l]}\left(C^{i}\left[F_{k}(H_{1}(x))\right]\right)⊕\left({⊕}_{j\in [n-l-1]}\left(C^{j}\left[F_{k}(H_{1}(x))\right]\right)\right)\in \left({⊕}_{i\in [t]}\left(C^{i}\left[F_{k}(H_{1}(Q))\right]\right)\right)$$⊕\left({⊕}_{j\in [n-l-1]}\left(C^{j}\left[F_{k}(H_{1}(Q))\right]\right)\right)$. Therefore, we need only think about $x\in X_{n}\backslash Q$. For all $x\in X_{n}$, $A\left[F_{k}(H_{1}(x))\right]={⊕}_{i\in [l]}\left(C^{i}\left[F_{k}(H_{1}(x))\right]\right)⊕\left({⊕}_{j\in [n-l-1]}\left(C^{j}\left[F_{k}(H_{1}(x))\right]\right)\right)$, the output of Hyb5 changes only if there exist $x\in X_{n}\backslash Q$, $y\in Q_{2}$ satisfying $y={⊕}_{i\in [l]}\left(C^{i}\left[F_{k}(H_{1}(x))\right]\right)⊕\left({⊕}_{j\in [n-l-1]}\left(C^{j}\left[F_{k}(H_{1}(x))\right]\right)\right)$.
  Suppose there is a PPT adversary A that, with non-negligible probability, produces Q, $Q_{2}$, and $X_{n}$ such that there exist $y\in Q_{2}$, $x\in X_{n}\backslash Q$ satisfying $y={⊕}_{i\in [l]}\left(C^{i}\left[F_{k}(H_{1}(x))\right]\right)⊕\left({⊕}_{j\in [n-l-1]}\left(C^{j}\left[F_{k}(H_{1}(x))\right]\right)\right)$. Then, [5] shows we can break the security of the PRF.
Hyb6 Same as Hyb5 except that the protocol terminates if there exists $x^{a}\in Q$, $x^{b}\in X_{n}$ such that, $y={⊕}_{i\in [l]}\left(C^{i}\left[F_{k}(H_{1}(x^{a}))\right]\right)⊕\left({⊕}_{j\in [n-l-1]}\left(C^{j}\left[F_{k}(H_{1}(x^{a}))\right]\right)\right)=A\left[F_{k}(H_{1}(x^{b}))\right]$ but $x^{a}\neq x^{b}$. The protocol is aborted with negligible probability because of the security of the PRF.
Hyb7 Same as Hyb6 except that $P_{n}$’s outputs are substituted by its outputs in the ideal world. Hyb7 can change $P_{n}$’s outputs if and only if there exists a value φ received by $P_{n}$ and considered by $P_{n}$ such that $\varphi =H_{2}\left({⊕}_{i\in \left[l\right]}\left(C^{i}\left[F_{k}(H_{1}(x^{a}))\right]\right)⊕\left({⊕}_{j\in \left[n-l-1\right]}\left(C^{j}\left[F_{k}(H_{1}(x^{a}))\right]\right)\right)\right)$ for some $x^{a}\in Q$, and $\varphi =A\left[F_{k}(H_{1}(x^{b}))\right]$ for some $x^{b}\in X_{n}$, $x^{a}\neq x^{b}$. Because $H_{2}$ is a random oracle, ${⊕}_{i\in [l]}\left(C^{i}\left[F_{k}(H_{1}(x^{a}))\right]\right)⊕\left({⊕}_{j\in [n-l-1]}\left(C^{j}\left[F_{k}(H_{1}(x^{a}))\right]\right)\right)\neq A\left[F_{k}(H_{1}(x^{b}))\right]$ is aborted via terminate condition in Hyb6 with negligible probability.
Hyb8 Same as Hyb7 except that the protocol does not terminate. Hyb7 and Hyb8 are computationally indistinguishable since $H_{1}$ and $H_{2}$ are random oracles and $F_{k}$ is a PRF.
Hyb9 The output in the ideal world. The difference between Hyb9 and Hyb8 is that S samples a random matrix C and encodes a data structure PaXoS, which is identically distributed.

□

## 5. Performance Evaluation

### 5.1. Complexity Analysis

To better evaluate the complexity of the protocol, we first need to perform a simple analysis of the overall protocol process. It is important to note that this protocol uses only inexpensive tools such as OTs and bitwise operations, making it concretely efficient. We treat t as the set sizes and set m=t as in [5]. So, w can be viewed as a value dependent on λ by fixing m and t.

Party $P_{n}$ is referred to as the leader carrying the majority overhead of the protocol, while the others are referred to as clients. Regarding the complexity of the protocol, $P_{n}$ designs matrices of a particular form, requiring linear complexity in t. Then, they perform w OTs for clients independently, resulting in linear complexity in the number of OTs. Moreover, $P_{i\in [n-2]}$ just do encoding operations for data structure $D^{i}$, and $P_{n-1}$ does hashing, bitwise-XOR, and decoding operations, which require linear communication and computation complexities. Although the computational overhead of $P_{n-1}$ is larger than that of other clients, they do not need to encode and send a data structure. From this, we can regard the overall communication and computation costs as uniformly distributed across all clients.

Note that our protocol can be divided into offline and online phases. Only lightweight procedures are required in the online phase, and communication and computation costs associated with performing OT can be handled in the offline phase. In addition, the bits exchanged among the parties concerning the random OT and the optimized malicious OT extension are summarized in Table 3.

### 5.2. Comparison

It should be noted that, due to the variations in architectures and security levels, making a fair comparison is challenging. Nevertheless, we have endeavored to include some recent studies pertaining to diverse security models (e.g., semi-honest, malicious, etc.). So, we contrast the complexity of communication and computation with [13,14,15] in Table 4, where n is the number of parties, k is the number of hash functions, t is the size of input sets, and λ is the security parameter. In our MPSI protocol, the communication and computation complexity of the leader are O(tnλ), which is linear in the number of parties. Meanwhile, the complexity for the client remains constant regardless of the number of parties involved (namely, O(tλ)) because the client $P_{i\in [n-1]}$ only needs to compute and send a data structure $D^{i}$ and does not need to perform additional data transfers with other parties. Therefore, our protocol achieves a good trade-off between communication and computation overhead.

**Figure 5:**
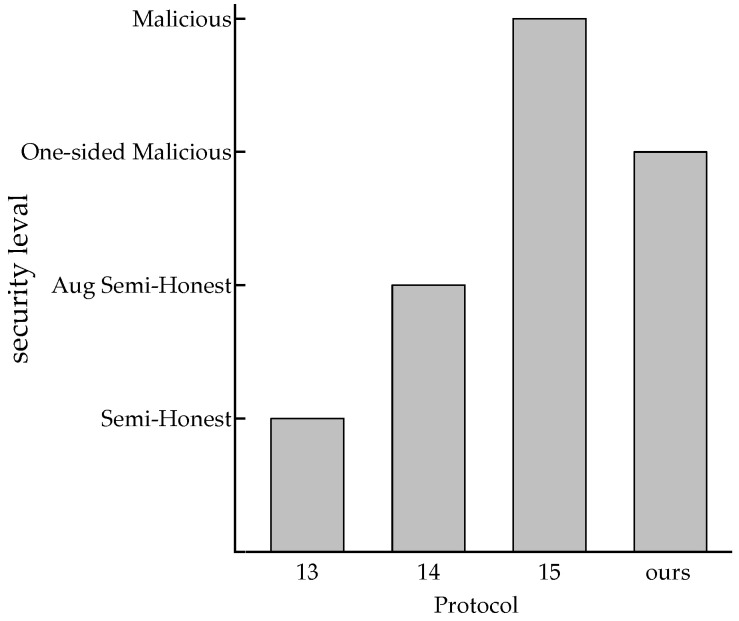
Comparison of security levels.

Figure 5 shows the security levels of the discussed protocols. Compared with [13], our protocol achieves a stronger security model without sacrificing communication and computation costs. We implement one-sided malicious security and [14] implements the Aug semi-honest model. It is difficult to define which security model is more practical, but our protocol has better computation and communication performance. Although the security model in [15] is higher-performing, our protocol has greater communication performance and achieves a better trade-off between communication and computation.

### 5.3. Experimental Evaluation

In order to compare the runtime overhead of each protocol more intuitively, simulation experiments and a results analysis were performed. It should be noted that the time consumed by this protocol is the average time of multiple experiments. The experimental platform was Windows 10, Intel (R) Core (TM) i5-8250U CPU @ 1.60 GHz 1.80 GHz, 8.00 GB of RAM, and a compiled environment of Dev-C++5.11.

**Figure 6:**
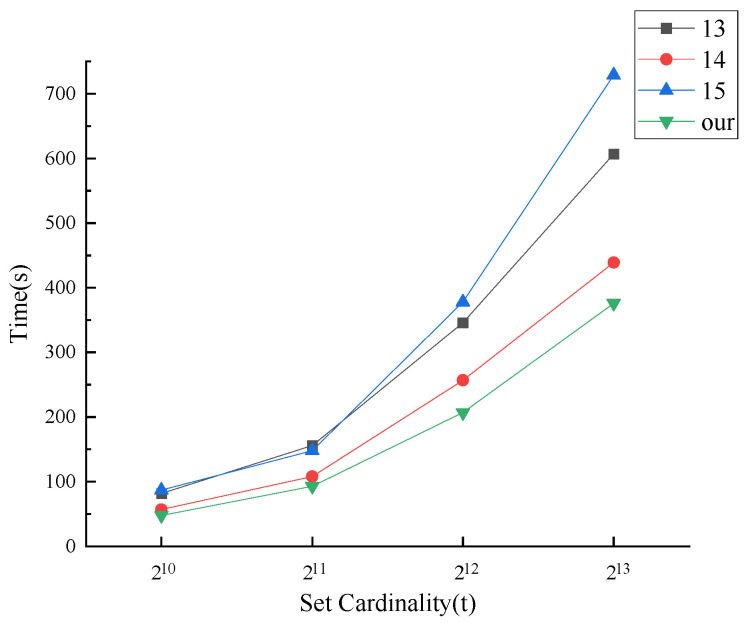
Running time vs. set cardinality.

We first consider the total time required for each protocol to execute with different numbers of set elements. It is assumed that n=100, k=λ=128, and $t=2^{10},2^{11},2^{12},2^{13}$ are chosen for the comparison experiment, and Figure 6 shows the total running time of the protocol as a function of the number of elements contained in the set.

From Figure 6, the total time overhead in each protocol grows essentially linearly as the number of set elements continues to increase. However, the time of our MPSI protocol increases the slowest when the fixed set cardinality is small. Our MPSI protocol has the slowest time growth rate.

**Figure 7:**
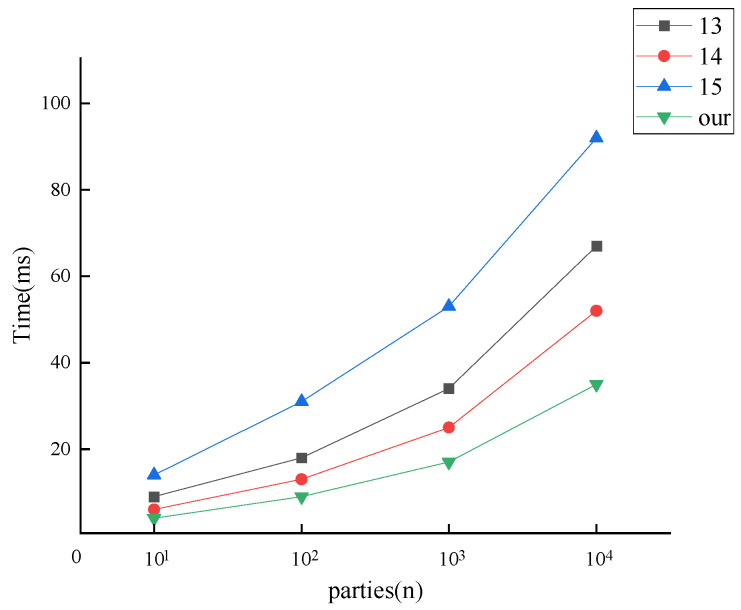
Running time vs. the number of parties.

In addition, the effect of the change in the number of parties on the running time of the protocol is further considered. Suppose that the maximum number of elements contained in the set is t=1000, the security parameters are kept fixed at k=λ=128, and the number of parties $n=10^{1},10^{2},10^{3},10^{4}$ is selected for the comparison experiment. The total protocol runtime as a function of the number of parties is shown in Figure 7.

From Figure 7, the running time of all protocols increases gradually with the number of parties. The time overheads of our MPSI protocol are lower than those of the other protocols when n is fixed. In addition, our MPSI protocol has the slowest time growth rate.

## 6. MPCCache in Edge Computing

**Figure 8:**
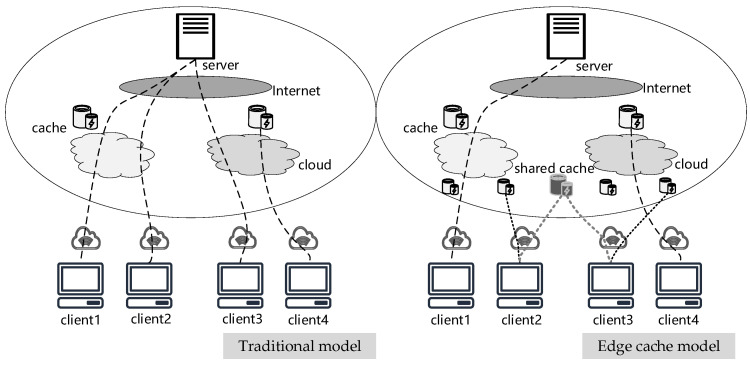
Traditional cache model and edge cache model.

This section aims to address the problem of edge collaborative content caching, wherein all parties can jointly cache the most frequently accessed common data items in shared caches. Figure 8 shows the difference between the traditional cache model and edge cache model. Our challenge is to find how to determine a set of the most frequently accessed common items without revealing any underlying data.

### 6.1. Our MPCCache

We describe how to use our MPCCache protocol to handle the edge cache case. The network operators $P_{i\in [n]}$ respectively own set $K_{i}=\{(x_{1}^{i},z_{1}^{i}),\ldots ,(x_{t}^{i},z_{t}^{i})\}$, where $x^{i}\in {\left\{0,1\right\}}^{*}$ denotes an identify element and $z^{i}\in {\left\{0,1\right\}}^{w}$ denotes its associated value. Note that the latter may represent the anticipated frequency of content being accessed or the value to network operators of the cached content. Let the common items $I=\cap_{i=1}^{n}X_{i}=\{x_{1},x_{2}\ldots \}$ be the intersection of the identifiers, where $X_{i}=\{x_{1}^{i},\ldots ,x_{t}^{i}\}$ is the set of identity for $P_{i\in [n]}$. For each common item x∈I, calculate a sum of the associated values z; that is, $sum^{\left(x\right)}=\sum_{i=1}^{n}z^{\left(x\right)}$. The sum of a common item is determined as the total of the individual values of the operators for the item.

**Figure 9:**
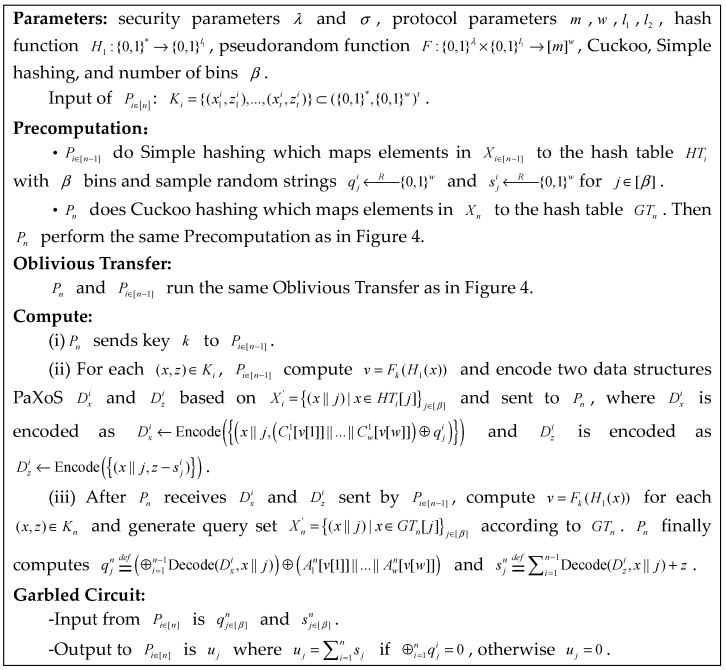
Our MPCCache protocol.

We present the MPCCache protocol in Figure 9. $P_{i\in [n-1]}$ conduct simple hashing and $P_{n}$ conducts cuckoo hashing that maps common items to the same bucket. According to the PaXoS, all the buckets are compressed into a data structure so that $P_{n}$ can efficiently compute the MPCCache. In detail, $P_{i\in [n-1]}$ choose $q_{j}^{n}$ and $s_{j}^{n}$ uniformly at random for j∈[β]. Notice that $A_{j}^{n}[v[j]]={⊕}_{i=1}^{n-1}C_{j}^{i}[v[j]]$ for each x∈I. For $(x,z)\in K_{i}$ and $v=F_{k}(H_{1}(x))$, $P_{i\in [n-1]}$ compute $f_{x_{j}}^{i}\overset{def}{=}\left(C_{1}^{1}[v[1]]||,\ldots ,||C_{w}^{1}[v[w]]\right)⊕q_{j}^{i}$ and $g_{x_{j}}^{i}\overset{def}{=}z-s_{j}^{i}$, and send the encoding $\mathrm{Encode}(x||j,f_{x_{j}}^{i})$ and $\mathrm{Encode}(x||j,g_{x_{j}}^{i})$ to $P_{n}$, where $x_{j}=(\mathrm{HT}[j]||j)$ means that x is in j−th bucket. $P_{n}$ can use $x_{j}^{n}\in {\left\{\left(x||j)|x\in {\mathrm{GT}}_{n}[j\right]\right\}}_{j\in [\beta ]}$ to obtain the correct decoding $f_{x_{j}}^{i}$ and $g_{x_{j}}^{i}$ if $x_{j}^{n}=x_{j}^{i}$; it is otherwise random. Then, $P_{n}$ computes $q_{j}^{n}\overset{def}{=}{⊕}_{i=1}^{n-1}f_{x_{j}}^{i}⊕\left(A_{1}^{n}[v[1]]||\ldots ||A_{w}^{n}[v[w]]\right)$ and $s_{j}^{n}\overset{def}{=}\sum_{i=1}^{n-1}g_{x_{j}}^{i}+z$. Finally, $P_{i\in [n]}$ input $q_{j\in [\beta ]}^{n}$ and $s_{j\in [\beta ]}^{n}$, respectively, to check whether ${⊕}_{i=1}^{n}q_{j}^{i}=0$ is based on a garbled circuit, and, if so, obtain the sum of the corresponding common item ${⊕}_{i=1}^{n}s_{j}^{i}$.

### 6.2. Correctness and Security

Correctness: Section 4.3 proves that $A_{j}^{n}[v[j]]={⊕}_{i=1}^{n-1}C_{j}^{i}[v[j]]$ for each x∈I and $v=F_{k}(H_{1}(x))$; that is, $A_{1}^{n}[v[1]]||\ldots ||A_{w}^{n}[v[w]]={⊕}_{i=1}^{n-1}\left(C_{1}^{1}[v[1]]||\ldots ||C_{w}^{1}[v[w]]\right)$. Via the property of the data structure PaXoSs $D_{x}^{i}$ and $D_{z}^{i}$ constructed by $P_{i\in [n-1]}$, for x∈I, j∈[β], and $v=F_{k}(H_{1}(x))$, we always have ${⊕}_{i=1}^{n-1}\mathrm{Decode}(D_{x}^{i},x||j)={⊕}_{i=1}^{n-1}\left(\left(C_{1}^{i}[v[1]]||\ldots ||C_{w}^{i}[v[w]]\right)⊕q_{j}^{i}\right)$, ${⊕}_{i=1}^{n-1}\mathrm{Decode}(D_{z}^{i},x||j)=\sum_{i=1}^{n-1}\left(z^{i}-s_{j}^{i}\right)$. At the same time, $P_{n}$ defines $q_{j}^{n}\overset{def}{=}\left({⊕}_{i=1}^{n-1}\mathrm{Decode}(D_{x}^{i},x||j)\right)⊕\left(A_{1}^{n}[v[1]]||\ldots ||A_{w}^{n}[v[w]]\right)$ and $s_{j}^{n}\overset{def}{=}\sum_{i=1}^{n-1}\mathrm{Decode}(D_{z}^{i},x||j)+z$ in terms of the $D_{x}^{i}$ and $D_{z}^{i}$ they receive from $P_{i\in [n-1]}$. That is, when x∈I, it always satisfies that ${⊕}_{i=1}^{n}q_{j}^{i}=0$ and ${⊕}_{i=1}^{n}s_{j}^{i}=\sum_{i=1}^{n}z^{i}$.

**Theorem** **2.** *If* F *is a PRF and* $H_{1}$ *is a random oracle, then the construction of our MPCCache protocol has colluding semi-honest security, given the OT, PaXoS, GC, and appropriate parameters.*

**Proof** **of** **Theorem** **2.** If we consider l parties ${\left\{P_{i}\right\}}_{i\in [l]}$ to be corrupted by an adversary A, then the number of uncorrupted parties is (n−l). Given ${\left\{K_{i}\right\}}_{i\in [l]}$, the simulator S interacts with ${\left\{P_{i}\right\}}_{i\in [l]}$ as follows. S samples random matrices, performs OT, chooses the PRF key k and sends k to ${\left\{P_{i}\right\}}_{i\in [l]}$. The simulator S constructs random data structures representing honest parties according to the randomness of the matrices. S sends two data structures $D_{x}^{i}$ and $D_{z}^{i}$ constructed on a PaXoS to ideal functionality. We prove ${\mathrm{Real}}_{A}^{\prod }\left(K_{1},\ldots ,K_{n}\right)\overset{c}{\approx }{\mathrm{Ideal}}_{S}^{F}\left(K_{1},\ldots ,K_{n}\right)$.

Hyb0 The outputs of parties in the real world.
Hyb1 Same as Hyb1, Hyb2, and Hyb6 in Section 4.4.
Hyb2 Similar to Hyb1 except that the decoding executions of the PaXoS are replaced as follows. When ${\left\{P_{i}\right\}}_{i\in [l]}$ does not contain $P_{n}$, S receives nothing from the data structure PaXoS. When ${\left\{P_{i}\right\}}_{i\in [l]}$ contains $P_{n}$, if x∈I, $P_{n}$ receives $D_{x}^{i}$ and $D_{z}^{i}$, thus $\left(C_{1}^{i}[v[1]]||\ldots ||C_{w}^{i}[v[w]]\right)⊕q_{j}^{i}$, $\left(z^{i}-s_{j}^{i}\right)$ for the PaXoS involving the non-colluding party ${\left\{P_{i}\right\}}_{i\in [n-l]}$ and j∈[β]. Note that $q_{j}^{i}$ and $s_{j}^{i}$ are used in the above expression for each bin j∈[β]. Since these values are uniform, so are $D_{x}^{i}$ and $D_{z}^{i}$. Therefore, we replace the decoding outputs of the PaXoS with random ones. Otherwise, all the decoding outputs of the PaXoS are uniformly random from the perspective of $P_{n}$ and ${\left\{P_{i}\right\}}_{i\in [l]}$. Hyb2 is computationally indistinguishable from Hyb1 due to the PaXoS’s security.
Hyb3 The output in the ideal world. The only difference between Hyb3 and Hyb2 is that S executes the output of the circuit.

□

## 7. Conclusions

In this work, we design an efficient MPSI protocol and the MPCCache protocol to better solve the information leakage problem in resource sharing. The proposed MPSI protocol derived from multi-point OPRF demonstrates concrete efficiency in achieving one-sided malicious security. The protocol also leads to a better trade-off between communication and computational overhead. It is based on OT and a data structure PaXoS and achieves linear computation and communication complexity concerning the input set size of each party. In our MPSI protocol, the asymptotic communication and computational complexity of the clients are largely determined by the size of the input sets rather than the number of parties (namely, O(tλ)). Overall, this research has contributed to the development of efficient MPSI protocols for multiple parties in practice. In fact, we apply the MPCCache protocol to edge caching scenarios using a simple transformation of the MPSI protocol. The MPCCache protocol under the semi-honest model can support the computation of specific functions on intersections. It is our belief that future work can improve the fairness of the MPSI protocol, as well as propose more application scenarios with practical application value.

## Figures and Tables

**Table 1:** The related work of PSI.

Protocol	Technical	Number of Parties	Security Model
[15]	OLE	multi-party	Malicious
[16]	OT	two-party	Semi-Honest
[17]	GBF + OT	two-party	Semi-Honest
[18]	OPPRF + OKVS	multi-party	Malicious
[19]	PaXoS	two-party	Malicious
[20]	GBF	multi-party	Malicious
[21]	PCG	two-party	Semi-Honest

**Table 2:** The related work of function-based PSI.

Protocol	Technical	Number of Parties	Protocol Type
[3]	DDH + HE	two-party	PI-Sum
[4]	OPPRF	multi-party	MPCCache
[11]	OPPRF + Circuit	multi-party	PSI- payload
[22]	OPRF + DDH	two-party	PIW-Sum
[23]	DOPRF	two-party	PSI-CA

**Table 3:** Bits sent for leader and client.

Communication Party	Total Bit Transmission
$P_{n}\to P_{i\in [n-1]}$	tw
$P_{i\in [n-1]}\to P_{n}$	$w\left(\lambda -1\right)$
$P_{i\in [n-2]}\to P_{n-1}$	tw
$P_{n-1}\to P_{n}$	$tl_{2}$

**Table 4:** Complexity of MPSI protocols.

Protocol	Communication		Computation		Security Model
	Leader	Clients	Leader	Clients	
[13]	O(tnλ)	O(tλk)	O(tnλ)	O(tnλ)	Semi-Honest
[14]	$O\left(\log(n)tn\lambda k\right)$	$O\left(\log(n)tn\lambda k\right)$	$O\left(tn\lambda k\right)$	$O\left(tn\lambda k\right)$	Aug Semi-Honest
[15]	$O((n^{2}+tn)\lambda )$	O(tλ)	O(tnlog(t))	O(tlog(t))	Malicious
Ours	O(tnλ)	O(tλ)	O(tnλ)	O(tλ)	One-sided Malicious

## Data Availability

Not applicable.
